# Passage- and serum-dependent changes of adipose-derived stromal cells *in vitro*: a discrepancy of stemness factors regarding mesenchymal surface markers and expression of stemness-related genes

**DOI:** 10.1515/iss-2024-0010

**Published:** 2024-12-02

**Authors:** Renata Sonnenfeld, Peter M. Vogt, Jörn W. Kuhbier, Sarah Strauss

**Affiliations:** Department of Plastic, Aesthetic, Hand and Reconstructive Surgery, Hannover Medical School, Hannover, Germany; Department of Palliative Care, University Hospital Basel, Basel, Switzerland; Department of Plastic, Aesthetic, Hand and Reconstructive Surgery, Helios Klinikum Hildesheim, Hildesheim, Germany

**Keywords:** human adipose-derived stem cell, stemness capacity, tissue engineering, stem cell niche, culture protocol, fat transplantation

## Abstract

**Objectives:**

Autologous fat transplantation is a promising source for cell therapy and tissue engineering. However, the physiological function and regulatory mechanisms of *in vitro* cell culture remain largely unexplored. Furthermore, no standard protocol for cell culture of human adipose-derived stem cells (hASC) has been described. Previous studies have reported the impact of media supplementation on the loss of stemness capacity.

**Methods:**

In this study, we compared the expression of stemness-defining surface markers according to the minimal criteria definition (CD 11b⁻, CD 31⁻, CD 34⁻, CD 45⁻, CD 73⁺, CD 90⁺, and CD 105⁻) by flow cytometry analysis with the expression of stemness-related genes such as MCAM, OCT4, MYC, and cKit in hASCs cultured in either fetal calf serum (FCS) or human serum (HS) supplemented medium from passage 0 to 5.

**Results:**

As expected, we found that hASCs in both groups retained their typical mesenchymal surface marker profile CD 73 and CD 90 (>95 %) in flow cytometry analysis, as well as the absence of CD 11b, CD 31, CD 34, and CD 45 (<5 %) until passage 5. However, in contrast to that, RT-PCR indicated a passage-dependent decline and medium-dependent changes in the transcriptome, in particular the loss of the stemness-related genes cKit and MCAM in both groups, while MYC and OCT4 showed unpredictable expression.

**Conclusions:**

Summarized, these results indicate the need for standardized cell culture protocol, as the transcriptome seems to change during *in vitro* cultivation, although an ASC-typical pattern of surface markers remains. In this regard, our study aims to contribute to the establishment of a standard protocol to achieve reliability, validity, and objectivity for future cell therapy or clinical applications.

## Introduction

Since the isolation and characterization of human adipose-derived stem cells (hASCs), new applications and cell-based therapeutic options have been widely explored. The advantages of hASC harvest, such as accessibility and minimal donor side morbidity, as well as their abundance in subcutaneous adipose tissue, are striking and already numerously described [1], [2], [3], [4], [5], [6], [7]. As mesenchymal stem cells, they are capable of multilineage differentiation, immunomodulation, antiapoptosis, angiogenesis, and self-renewal [1], [2], [3], [4]. As a promising source for cell-based therapies, hASCs have been successfully used in ischemic revascularization, cardiovascular tissue regeneration, soft tissue augmentation, and reconstruction [8], [9], [10]. Their characteristic properties are due to their stemness capacity, which is preserved *in vivo* by their microenvironment, the so-called stem cell niche. Currently, the phenotypic change of stemness during *in vitro* culture remains unpredictable and, therefore, a disadvantage or even a risk for the clinical use of hASCs. Mimicking the physiological conditions, the so-called stem cell niche, in *in vitro* cell culture is a delicate task and not yet fully understood. Cultivation protocols still vary with regard to the amount of serum supplementation. We used 20 % serum supplementation in our media, whereas the most common serum supplementation in the literature is 10–20 %. We chose a higher percentage because a large amount of research data has shown that the use of higher serum concentrations increases proliferation rate and cell expansion [11], 12]. Often a high number of cells is required for certain applications, unmentioned due to loss of donor substance in the transplantation process. For regenerative medicine applications such as transplantation or gene therapy, autologous hASCs need to be expanded *in vitro* and then reimplanted into the target tissue [13]. The current standard use of fetal calf serum in cell culture carries a risk of immune reactions and infection and indeed remains unpredictable in terms of recipient safety [14]. Previous studies have demonstrated that replacing bovine serum by allogeneic human serum is beneficial for cell proliferation [15], 16]. Thorough characterization of hASCs is essential prior to their clinical application in patients. On the one hand, we investigated stemness by the expression of a typical pattern of surface markers (CD 73⁺, CD 90⁺, CD 11b⁻, CD 31⁻, CD 34⁻, CD 45⁻) using flow cytometry according to the minimal criteria definition and the joint statement of the International Federation for Adipose Therapeutics and Science (IFATS) with the International Society for Cellular Therapy (ISCT) [17]. On the other hand, we investigated the same cells regarding stemness-related genes via RT-PCR. With this study, we aim to gain further knowledge to describe and preserve the stemness capacity by improving the cell culture conditions to mimic physiological *in vivo* conditions.

## Materials and methods

### Isolation and cell culture

After receiving approval from the Ethics Committee of Hannover Medical School (reference no. 1569-2012) and the donors’ signed written consent, human adipose tissue was harvested from elective dermolipectomy and the stromal vascular fraction containing adipose-derived stem cells was isolated. The donors (n=5; male=2, female=3) had no significant medical history other than obesity and were aged between 28 and 55 years (mean=35.6 years). After the surgical procedure of dermolipectomy, the fat tissue was removed from the graft, minced, and transferred into sterile multipurpose screw-capped cups (size 50 mL). Each cup was filled with a 20 mL fraction of minced fat and 0.2 % (w/v) of collagenase (C1-22, Biochrom) in Hank’s buffered salt solution to digest the extracellular matrix. The sample was then vortexed and incubated for 1 h at 37 °C with gentle motion agitation at 120 rpm. The suspension was then dispersed, washed with Hank’s buffered salt solution, and centrifuged for 5 min at 30 ×*g*. The oily upper fraction and the pellet at the bottom were transferred and filled with Hank’s buffered salt solution and 2 g/L bovine serum albumin and centrifuged for 10 min at 300 ×*g*. The liquid fraction was discarded, and the pellet was resuspended with Hank’s buffered salt solution, followed by centrifugation for 10 min at 300 ×*g* and siphoning of the liquid fraction. Each pellet, containing the stromal vascular fraction, was then resuspended in a 150 cm^2^ flask (TPP 90151, Sigma Aldrich, Munich, Germany) with 20 mL DMEM/F-12 (Biochrom, FG 4815; Berlin, Germany) contained either 20 % fetal calf serum (FCS; Biochrom, S0615; Berlin, Germany) or 20 % human serum. Apart from the different serum, both media contained similar 50 U/mL penicillin/streptomycin (Biochrom, A2212; Berlin, Germany), 0.173 mM ascorbate-2-phosphate (Sigma Aldrich, A8960; Munich, Germany). Flasks were incubated at 37 °C, 5 % CO_2_ and water-saturated atmosphere. Cultures were detached with 0.25 % Trypsin-EDTA at approximately 70 % confluence, split 1:3 and passaged to passage 5.

### Human serum preparation protocol

Human serum was obtained from residual blood samples of healthy donors from the Hannover Medical School Blood Bank. In the donation procedure, a small amount of donor blood often remains unused and is usually discarded. These residuals were collected anonymously in tubes and allowed to clot. After 30–60 min at room temperature, the tubes were centrifuged at 2000 ×*g* for 10 min to separate the serum from the clot. The serum was immediately pipetted into new tubes and heat-inactivated in a water bath at 56 °C for 30 min. The pooled mixed-gender serum was stored at −80 °C until use.

### Metabolic activity

To investigate the effect of media on viability, we performed CellTiter-Blue^®^ Cell Viability Assay (Promega, Fitchburg, USA) according to the manufacturer’s protocol. Fluorescence was measured using the Tecan GENios multi-well reader (Männedorf, Switzerland). About 500 cells per well were seeded on 96-multi-well plates. Both groups (HS, FCS) received daily changes of media and were incubated as described above. Cell viability was determined at 24, 48, 72, 96, 120, and 144 h. The assay is based on the metabolic function of viable cells to reduce the used dye into a fluorescent substrate, which can be detected by a spectrophotometer.

### Cell confluency

Cell cultures of 150 cm^2^ flask of both groups were monitored daily by light microscopy (Olympus CK-40, Shinjuku, Japan). Increasing confluence was photodocumented with 40× enlargement. At approximately 70 % confluence, cells were detached and passaged at a 1:3 ratio. We monitored the maintenance of the typical fibroblast-like morphology of the ASCs as well as the monolayer architecture and the cell density. The time to reach app. 70 % confluence (tc70) by subjective evaluation was documented and compared between both groups at each passage until P5. Media changes were performed three times per week when the color of the media indicated an acidic pH, using 20 mL of human-serum supplemented vs. fetal calf-supplemented media per 150 cm^2^ flask.

### Flow cytometry analysis

According to the minimal criteria definition for multipotent mesenchymal stem cells [4], we investigated the presence of specific surface markers CD 73 and CD 90 and the absence of CD 11b, CD 31, CD 34, CD 45, and CD 105 were examined by flow cytometry analysis from passage 0 to 5. The investigation was performed with a Beckman Coulter FC500 CellSorter (Brea, USA). The antibodies for CD 34 (Beckmann Coulter; A21691) and CD 73 (Biolegend; 344003) were PE-based/conjugated. The antibodies used for CD 31 (Beckmann Coulter; IM1431U), CD 45 (Beckmann Coulter; IM2652U), and CD 105 (Beckmann Coulter; PN A07414) were FITC-based/conjugated. The antibody used for CD 11b (eBioscience; 25-0118) was PC7-based/conjugated and that used for CD 90 (Beckmann Coulter; PN IM3703) was PC5-based/conjugated. Cells were detached at 70 % confluency for each passage. We incubated the cells with 10 µL of each antibody and 10 µL of the isotype for 45 min at room temperature in the dark. Each tube was washed with 1 mL of PBS and centrifuged at 300 ×*g* for 5 min. The liquid fraction was discarded, and the remaining cell pellet was suspended in 500 µL Isoton solution for immediate cytometry analysis.

### RT-PCR

Cells from P0-P5 were detached from the flasks when they reached approximately 70 % confluence, evaluated by light microscopy using 0.25 % Trypsin-EDTA (TE). After washing with PBS (wo), the cells were detached from the flask, centrifuged at 300 ×*g* for 5 min, and the enzymatic reaction was stopped by the addition of RA1-Lysis buffer. RNA isolation was performed with NucleoSpin RNAII-Kit (Macherey-Nagel, Düren, Germany) following the standard manual instructions. The quality and quantity of the extracted RNA was verified by using a NanoDrop 2000/2000c spectrophotometer (ThermoFisher, Waltham, Massachusetts, USA). RNA purity and concentration, as well as possible protein contamination, were tested before any further measurements. The integrity of the isolated RNA was verified by gel electrophoresis. cDNA synthesis was performed using the iScript^tm^ Synthesis Kit (BioRad, Hercules USA) according to standard manual instructions. RT-PCR was performed by using an iCycler real-time polymerase chain reaction cycler (BioRad Laboratories, Hercules USA). The genes analyzed were MCAM, cKIT, MYC, and OCT4 as shown in Table 1. All target gene expression levels were normalized with housekeeping genes (ywhaz, RPL 32, RPL 37, TBP, and β2-microglobulin) and quantified relative to the 2-ΔΔCT method [18]. Each RT-PCR plate layout included positive controls (pooled cDNA from N1-5 in P0, HPLC-water, EvaGreen, housekeeping gene-primer), negative controls (HPLC-water, EvaGreen, Housekeeping gene-Primer), and a blank control (HPLC-water and EvaGreen). The quality of selected primer pairs was validated by melting curve analysis and verification of product size on 2 % agarose gel electrophoresis (Biozym, 840004; Oldendorf, Germany).

**Table 1:** List of gene-specific PCR primers with forward (F) and reverse (R) sequences of primer pairs and accession number.

Gene	Primer sequence (5´ → 3′)	Accession no.
MCAM	F: ATCGCTGCTGAGTGAACCACAG (22)	NM_006500.3
	R: CTACTCTCTGCCTCACAGGTCA (22)	
cKIT	F: CACCGAAGGAGGCACTTACACA (22)	NM_000222
	R: TGCCATTCACGAGCCTGTCGTA (22)	
OCT4	F: CCTGAAGCAGAAGAGGATCACC (22)	NM_002701
	R: AAAGCGGCAGATGGTCGTTTGG (22)	
MYC	F: CCTGGTGCTCCATGAGGAGAC (21)	NM_001354870
	R: CAGACTCTGACCTTTTGCCAGG (22)	

### Statistical analysis

Statistical analysis was performed using SPSS 16.0 software. All measurement data were expressed as mean±standard error of the mean (SEM). Comparison between groups was performed using Student’s t-test. A p-value of less than 0.01 was considered to be statistically significant.

## Results

### Cultivation

**Figure 1:**
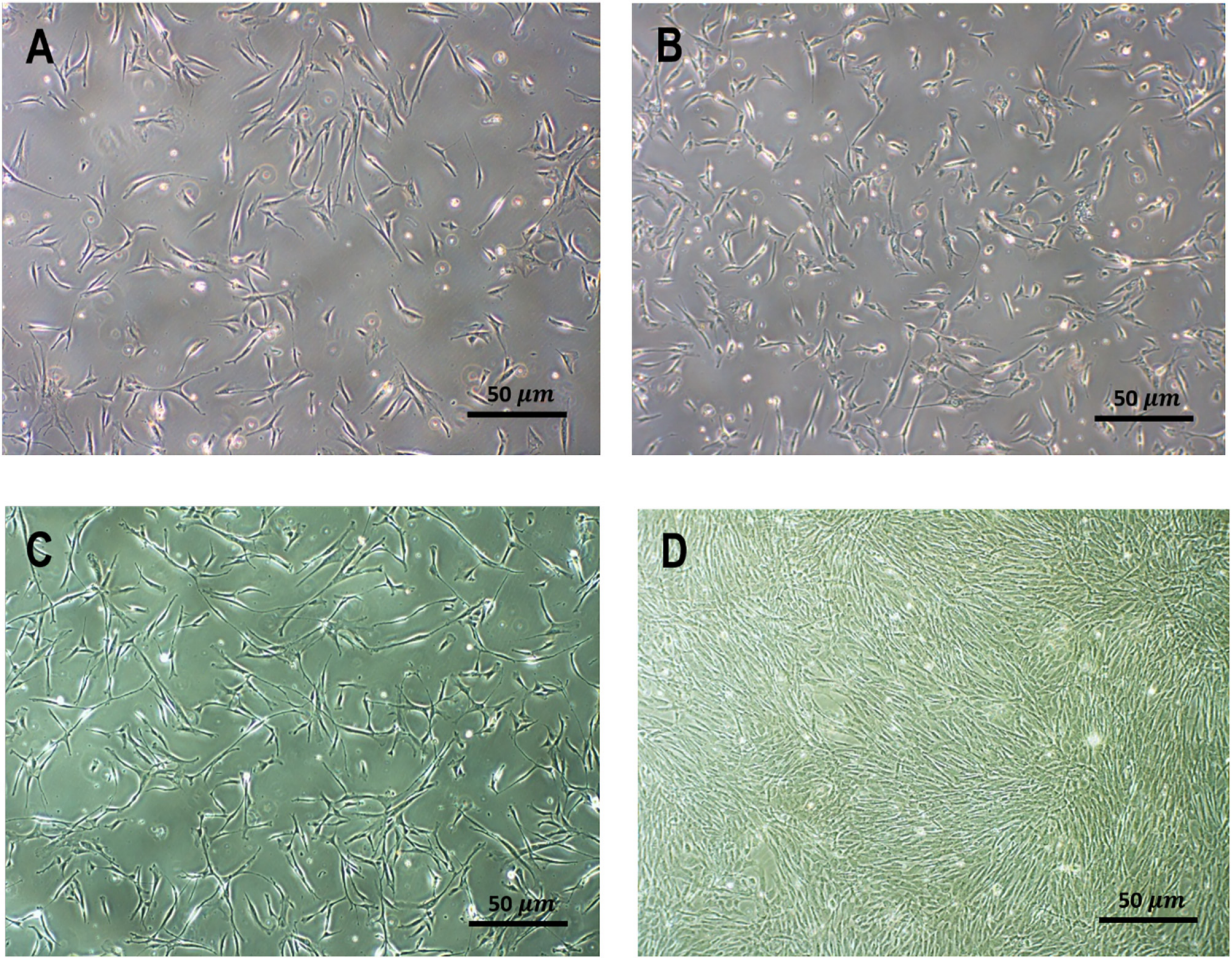
Typical fibroblast-like morphology and plastic adherence of hASCs from the same donor. (A) & (B) Day 5 after initial seeding (in P0) in different culture conditions: (A): 20 % FCS-supplemented media group, (B): 20 % HS-supplemented media group: a higher cell density can be seen in (B). Images (C) & (D) show the same hASCs in P2 15 days after initial seeding. (C): FCS group with app. 20 % confluence in P2, (D): HS group in P2 with app. >90 % confluence. An expansion of the monolayer structure was regarded as an increase in proliferation.

After the isolation process, the cell pellet, containing the stromal vascular fraction, was seeded into 150 cm^2^ flask, where the cells exhibited ASC typical growth behavior within 24 h. In both groups, cells developed a homogeneous monolayer with the characteristic fibroblast-like morphology and adherence to plastic. Furthermore, cell morphology and size remained unchanged throughout all passages up to P5 within all donor cells and both supplementation groups as shown in Figure 1.

### Cell viability

**Figure 2:**
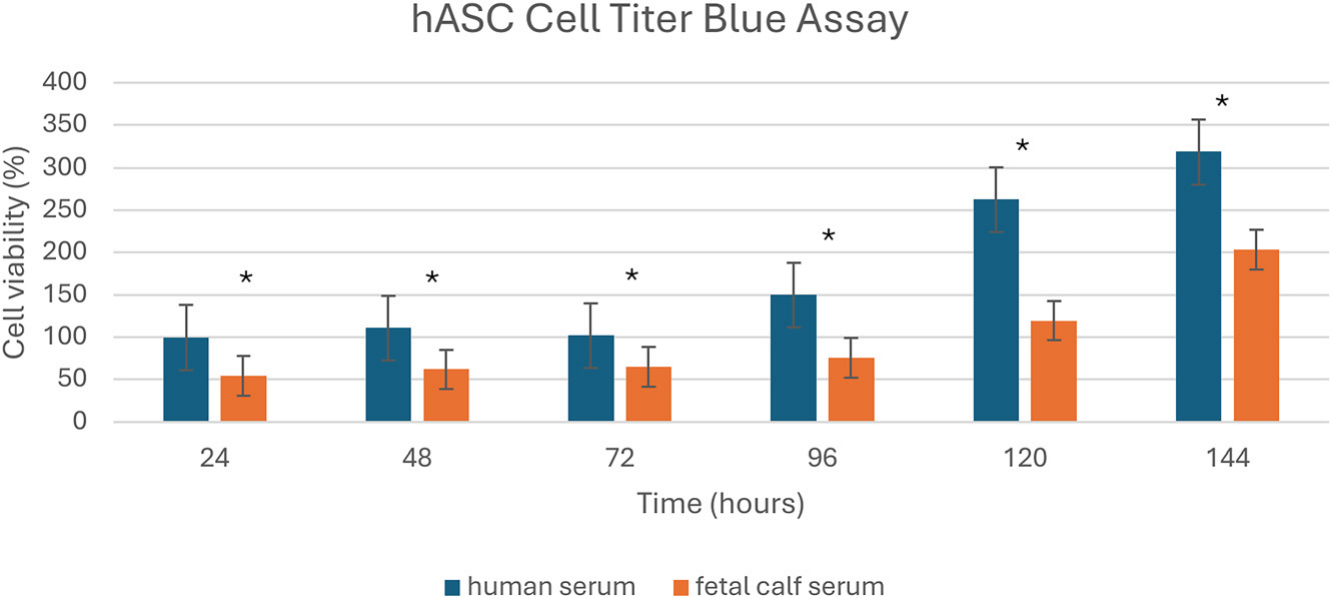
Cell viability evaluated by Cell Titer Blue assay. hASCs (P1) from the same donor were used in both groups (HS-supplemented and FCS-supplemented media). After 24, 48, 72, 96, 120, and 144 h metabolic activity was compared between both groups using Student’s t-test. hASCs in HS-supplemented media showed significantly higher cell viability at all times compared to hASCs cultured in FCS-supplemented media (*indicates statistical significance, p-value=<0.01). Relative cell viability was normalized to the highest viability achieved at 144 h in the HS supplemented media is shown.

As shown in Figure 2, metabolic activity was determined in both supplementation groups with hASCs from the same donor at passage 1 (P1) using the Cell Titer Blue assay. Significant differences (p-value=˂0.01 in student’s t-test) between the groups were already detectable after 24 h hASCs with HS supplementation displayed an almost 2-fold higher activity after 24 h and maintained their superiority throughout the measurement period. In the subsequent comparison of the FCS vs. HS-group, cell viability was significantly higher in the HS group at 24, 48, 72, 96, 120, and 144 h. The metabolic activity of the HS group was 2-fold higher than that of the FCS group at both 96 and 120 h. Viability, and therefore proliferation rate, remained significantly increased in human serum supplemented hASCs from day 1 (24 h) to day 6 (144 h).

### Cell confluency

**Figure 3:**
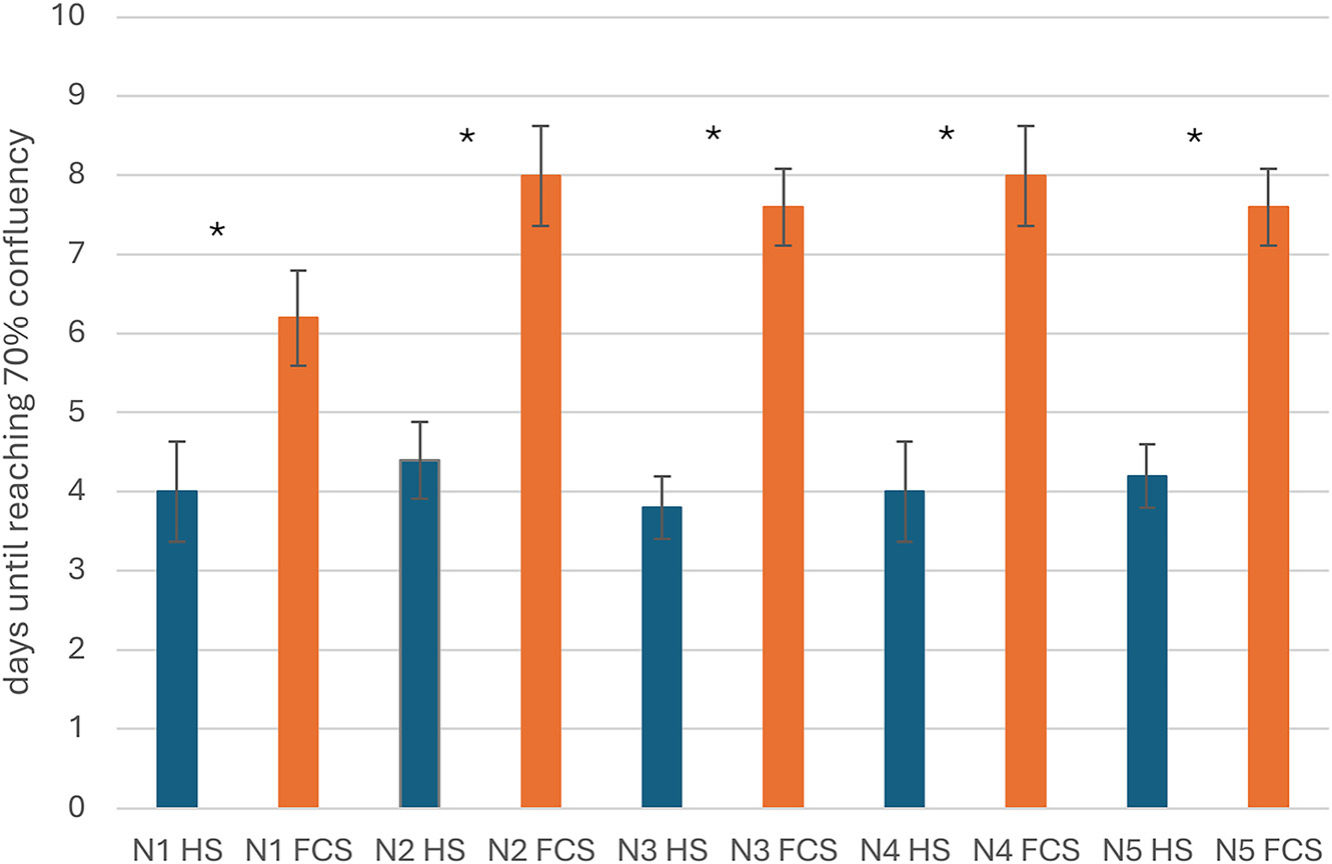
In addition, cell proliferation was determined as days to reach 70 % confluence. hASCs from 5 donors (N1-5) were examined through all passages (P0-P5) in both groups (HS vs. FCS supplemented media). Initial seeded cell number was 2 × 103 cells/cm^2^. The data show approx. 70 % confluence at a mean of 4.08 days (SEM±0.09) for hACS with HS-supplemented media, whereas hASCs in FCS-supplemented media had a mean of 7.49 days (SEM±0.29). Cell proliferation was compared in HS-group vs. FCS-group separately for each donor (N1-5) using Student’s t-test, revealing significantly higher cell proliferation in all HS-groups (*indicates statistical significance, p-value=<0.01).

While HS-supplemented hASCs reached monolayer confluence of 70 % at a mean of 4.08 days (SEM±0.09), hASCs in FCS-supplemented media had a mean of 7.49 days (SEM±0.29), as shown in Figure 3. The time to reach approximately 70 % confluence appeared to be constant and comparable for all hACS from 5 different donors (N1-5) through all passages (P0–P5) of both groups (HS vs. FCS supplemented media). The characteristic fibroblast-like morphology appeared unchanged. Consistent confluence assessment and cell documentation was performed using a light microscope with 50 µm enlargement (Olympus CK40, Tokyo, Japan) as shown in Figure 1. Adherent fibroblast-like shaped cells are shown in pictures A and B on day 5 after seeding of hASCs from the same donor (P0). Image A shows hASCs cultured in FCS-supplemented media; B shows the HS-supplemented group. A higher cell density can already be seen in B. Images C and D show the same hASCs in passage 2. Image C visualizes the approximately 20 % confluency in the FCS-supplemented group, while image D shows the HS-supplemented group with >90 % confluence. The cell flasks we used to image cell confluence were used solely for confluence documentation and discarded from further examination as 70 % confluence as defined in the study protocol.

### Expression of surface markers

Flow cytometry was used to determine the expression of the surface markers CD 11b, CD 31, CD 34, CD 45, CD 73, CD 90, and CD 105 in both groups (HS vs. FCS) at P0 and P5. There was no significant difference in surface marker expression related to either passage or supplementation. The absence of certain stemness-related surface markers such as CD 11, CD 31, CD 34, CD 45, and CD 105 was demonstrated by a percentage of ˂5 % and the presence of stemness-related surface markers such as CD 73 and CD 90 was >95 % (Table 2). The markers remained stable, and the standard error of the mean (SEM) showed homogeneity in the present data.

**Table 2:** Phenotypic characterization of hASCs at passage 0 (P0) and passage 5 (P5) cultured in FCS- or HS-supplemented media.

Antigen	P0 in FCS (mean±SEM)	P0 in HS (mean±SEM)	P5 in FCS (mean±SEM)	P5 in HS (mean±SEM)
CD 11b	0.5±0.2	0.6±0.1	0.5±0.2	0.2±0.1
CD 31	1.1±0.1	0.2±0.07	1±0.04	0.1±0.05
CD 34	3.4±0.2	3.2±0.2	2±0.3	2.7±0.3
CD 45	1.9±0.2	1.7±0.2	2.2±0.2	1.4±0.2
CD 73	98.7±0.5	99±0.4	98.9±0.5	99.3±0.4
CD 90	99.2±0.2	98.6±0.3	98.9±0.3	99±0.3
CD 105	5.2±1	3.6±0.4	4.5±0.7	4.9±0.7

Surface markers were determined by flow cytometry of 5,000 cells from pooled cells from 5 donors. Means±SEM are presented as percentage of cells positive for each surface marker measured by flow cytometry.

### Gene expression in RT-PCR

**Figure 4:**
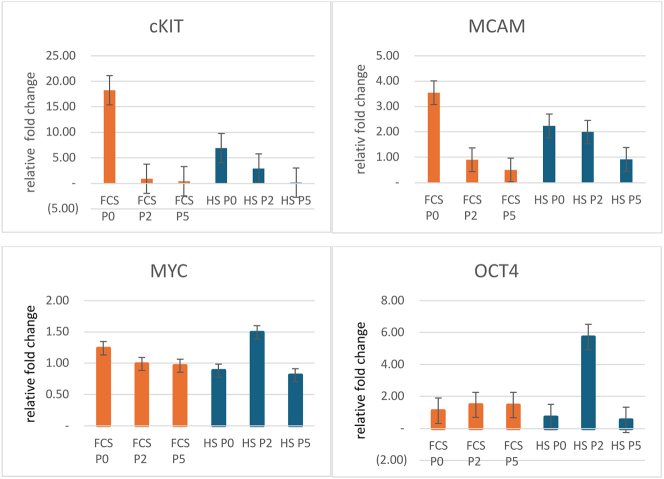
The relative change in the expression level of stemness-related genes (cKIT, MCAM, MYC, OCT4) in hASCs at P0, P2, P5 cultured in FCS-supplemented or HS-supplemented media. Pooled cells from 5 donors were evaluated. Means are expressed in relative units ±SEM. Statistical comparison between groups was performed using Student’s t-test; *p<0.01.

The expression of stemness-related genes, which are critical for the oncogenic transformation of human cells, was compared at P0, P2, and P5 by RT-PCR. Human cells were cultured in either FCS-supplemented or HS-supplemented media. Statistical comparison was made between conditions at the same time points. In both treatment groups, cKIT showed the highest downregulation (p<0.01) from P0 to P5. Similarly, MCAM expression decreased continuously during long-term culture from P0 to P5 (p<0.01). Interestingly, the initial (P0) expression levels of cKIT and MCAM were higher in FCS-treated cells compared to the initial levels in HS-treated cells. The expression levels of stemness-related genes are shown in Figure 4.

MYC and OCT4 showed no significant decrease from P0 to P5 in all treatment groups, although there was a slight tendency for MYC expression to decrease. When compared between passages P0 to P5, hASCs maintained in FCS-supplemented media had a stable level of OCT4 and MYC expression, whereas expression in HS group increased unpredictably at P2. However, treatment conditions (FCS vs. HS) had no significant effect on the changes in OCT4 and MYC gene expression.

Summarized, the data showed that certain regulatory genes (cKIT, MCAM) showed significant decrease in long-term culture from P0 to P5 in both treatment groups (FCS and HS). Yet notably, in all groups treated with FCS, gene expression levels were higher at P0, compared to P0 in HS media group.

## Discussion

The aim of the present study was to analyze differences in the maintenance of the stemness capacity of human adipose-derived stem cells in *in vitro* cell culture depending on passage number and serum supplementation. Serum supplements are commonly known to induce specific differentiation pathways in hASCs [1], [2], [3], [4], [5]. Multilineage capacity was a major and stemness-defining exploration by Zuk et al. in 2001 [1]. As various other research groups, Safwani et al. (2012) described a decrease of stemness-related genes in ASCs in long-term culture after osteogenic induction by media, we would like to test the hypothesis that the choice of serum supplement (human or calf) would also affect ASCs [19], 20].

### SVF

The isolated stromal vascular fraction consists of a heterogeneous cell population of ASCs, fibroblasts, preadipocytes, vascular endothelial cells, cytokines, growth factors, and yet only inadequately represents the diversity of the *in vivo* stem cell niche. Unlike the physiological niche, the SVF lacks key components such as cell-to-cell-contact, which can only be found within the original anatomical structures established by a heterogeneous population of progenitor and lineage-committed cells [21]. When cells are selected by the cell culture regime, in addition to ASCs, a selection of other adherent cohabitants of the stem cell niche, such as fibroblasts, are initially seeded. Thus, under *in-vitro*-conditions, cell–cell interaction, cell–extracellular matrix interaction, and secretion of signaling molecules secretion are lost as unique and important characteristics of the stem cell micromilieu [22].

### Stem cell niche vs. *in vitro* cell culture

Over the past decades, studies on bone marrow-derived stem cells have described the similar principles of a stem cell niche in the bone marrow and its relevance to the maintenance of stemness capacity within the cells. Schofield et al. described the interdependent homeostasis of the bone marrow stem cell niche as early as 1978 [23]. Their findings suggest that a specific milieu of cell–cell interactions, cytokine levels, and cell–extracellular matrix contacts is critical for the maintenance of stemness capacity of the mesenchymal stem cells. The preservation of the stem cell niche is mandatory for the regulation of stemness factors in the ACS. Moreover, it is key to regulatory processes in mesenchymal stem cells due to a plethora of cellular mechanisms [24], 25]. Voog et al. identified niches in various mammalian epithelial tissues [26]. Fuchs et al. described stem cell niches, e.g., in ovarian, testine, dermal, intestine, subventricular, and bone-marrow tissue in their review [27]. Stem cells in these niches have individual microenvironments that allow them to thrive and function as it is individually required [28], 29].

ASCs secrete a unique cytokine profile that allows them to induce cell proliferation and differentiation, promote angiogenesis, and reduce inflammation. In this regard, a state of constant calibration is required to maintain this delicate homeostasis. *In vivo*, this is regulated by the microenvironment of the ASC, the stem-cell niche. Alterations such as hypoxic conditions in cell culture would lead to distinct changes in the cytokine secretion profile. Rehman et al. investigated the secretion of angiogenic and antiapoptotic factors in ASCs and found that when ASCs were cultured under hypoxic conditions, e.g., vascular endothelial growth factor (VEGF) secretion increased 5-fold [5], 30].

Vice versa, hASCs maintained in coculture with other cells also affect these other cells in a variety of ways. For example, depending on the function of their neighbors, they may support hematopoiesis by releasing M-CSF *in vitro* in coculture with umbilical cord-derived CD34 cells. Furthermore, they seem to expand the amount of myeloid and lymphoid progenitor cells [6]. However, when maintained in coculture with T47D breast cancer cells, Kuhbier et al. (2014) revealed for the first time that ASCs fuse and initiate vesicular exchange with cancer cells [31]. Additionally, an increase in the expression of transcriptional genes for typical malignant markers was shown to be much higher in cocultures of ASCs and breast cancer cells than in single cultures [31].

### Stemness vs. malignancy

Since each stem cell divides into a progenitor cell and renews itself with each passage, it is understandable that the overall stemness capacity decreases overall during the time of cell cultivation. Progenitor cells lack multipotency and can only differentiate into one type of tissue, such as adipose tissue or osteoblasts. In addition, the renewed ASCs become more senescent with each cell division. Previous studies have shown that this leads to a dysregulation of proliferative activities and an overall decreasing stemness capacity [32]. These aged and dysfunctional stem cells accumulate toxic metabolites that lead to DNA damage and consequently the production of damaged proteins [33]. It is also shown in previous studies that the microenvironment surrounding the cells can either promote or retard cancer development [11], 25]. Plaks et al. described that stem cells that escape regulation within their niche can develop into a cancer stem cell [25]. Given their capacity to self-renewal and clonal expansion, the switch of a stem cell to its malignant counterpart leads to uncontrollable cancer development. Assuming similar mechanisms in the maintenance of stemness or deregulation *in vitro*, cell identification is as crucial as it is delicate. Prior to clinical use of ASCs expanded through multiple passages in cell culture for, the cells must be identified as nonmalignant to ensure a safe procedure.

### Surface markers and stemness-related genes

Regarding surface markers measured by flow cytometry, hASCs showed the required absence and presence of characteristic markers according to the original minimal criteria definition by Dominici et al. (2006) and the joint statement of the International Federation for Adipose Therapeutics and Science (IFATS) with the International Society for Cellular Therapy (ISCT) (2013) [4], 17]. However, RT-PCR showed a significant decrease in the stemness-related genes cKIT and MCAM from passage P0 to P5 in all treatment groups. More interestingly, and not yet described in previous studies, the initial levels of both markers were significantly higher at P0 in cells treated with FCS-supplemented media compared with cells cultured in HS. A decrease of these two stemness markers across all passages indicates a reduction in the signaling pathways involved in multipotency and consequently stemness capacity overall. Additionally, unpredictable variations in expression levels, such as those seen for OCT4 and MYC, indicate a currently unknown impact on the expression of regulatory genes within the cells. Moreover, transcriptional decrease of MCAM indicates loss of angiogenic function, as MCAM plays a fundamental role in mediating angiogenesis [34].

### Media influence on proliferation

Lindroos et al. reported that a higher percentage of human serum supplementation in the media results in a higher proliferation rate, which proportionally inclined due to the percentage ranging from 5 to 20 % [11]. However, these results were observed up to day 4 of cell culture. When the proliferation rate of ASCs in 20 % human serum supplemented media was compared with 10 % fetal calf serum media under the same culture conditions, it was equally high [11]. Our data on cell proliferation, as measured by cell viability showed comparable results in terms of the correlation between high serum percentages (20 %) and the attainment of app. 70 % confluence (FCS mean of 7.49 days (SEM±0.29), HS mean of 4.08 days (SEM±0.09)). It is noteworthy that ASCs cultured with human serum tend to grow faster than those cultured with fetal serum. A shorter *in vitro* cultivation time is beneficial not only for obvious reasons such as efficiency, but it also leads to less risks of contamination and loss of cells due to infection, simply because of the shorter *in vitro* exposure.

The stemness marker transcriptome shows variabilities from P0 to P5 with no predictable pattern in neither the HS-supplemented nor the FCS-supplemented group. Nevertheless, we found comparable pattern tendencies within some markers, such as cKIT and MCAM as well as OCT4 and MYC. cKIT and MCAM showed a constant decrease in expression in the FCS and HS cell population from P0 to P5.

### The impact of human serum

Human serum from blood donors contains a variety of allogenic growth factors, whereas the commonly used FCS is xenogenic to human ASCs due to its origin. However, the use of serum in cell culture is essential for the cell nutrition, and it promotes cell growth and consequently cell survival. Similar to other human blood derivatives, serum contains numerous growth factors to maintain ASC stemness, senescence, and differentiation and to promote overall growth [35]. Heat inactivation of human serum destroys complement activity but does not necessarily affect the growth properties and potential pathogens. Additionally, other protein components of the serum remain active and potentially harmful as pathogens of RNA or DNA origin [36]. This represents a potential risk of infection that can be considered a relevant uncertainty when using allogenic HS for human ACS cell culture.

Human blood donors and their blood samples must undergo an extensive screening process before being accepted for donation, so the risk of infection must be comparable to the general incidence of infection from blood transfusion in the country concerned. The same risks apply to FCS. However, to date there are no reliable data on the level or quality of infection in cell cultures due to the use of either FCS or HS.

Notably, a variety of advantages have been reported for the use of human serum in cell culture: Tallheden et al. showed that articular chondrocytes cultured in autologous human serum showed up to seven times higher proliferation than FCS-supplemented chondrocyte cultures [37]. Furthermore, Kobayashi et al. demonstrated that bone marrow derived MSCs cultured with autologous human serum exhibited greater cell motility than MSCs expanded in the presence of FCS [38].

## Conclusions

Our data support the notion that morphological characteristics and surface-marker expression do not necessarily correlate with stable transcriptome expression levels of regulatory genes in long-term culture of hASCs. Variable expression levels of regulatory and stemness genes in hASCs during long-term culture support this concern.

Regarding cell viability, both HS and FCS appeared to increase proliferation, but HS had a significantly stronger effect. In conclusion, based on the results of this study, we recommend the use of human serum in hASC cell culture for volume expansion.

Considering that there are still various isolation and cultivation protocols, there is a need to establish universal standard protocols to achieve reliability, validity, and objectivity in the overall use of adipose-derived stem cells. In conclusion, the need for standardized and uniform cell culture conditions is indispensable for the overall safety of cell therapy or applications.

## References

[1] Zuk PA, Zhu M, Mizuno H, Huang J, Futrell JW, Katz AJ (2001). Multilineage cells from human adipose tissue: implications for cell-based therapies. Tissue Eng.

[2] Zuk PA, Zhu M, Ashjian P, De Ugarte DA, Huang JI, Mizuno H (2002). Human adipose tissue is a source of multipotent stem cells. Mol Biol Cell.

[3] Kuhbier JW, Weyand B, Radtke C, Vogt PM, Kasper C, Reimers K (2010). Isolation, characterization, differentiation and application of adipose-derived stem cells. Biochem Eng Biotechnol.

[4] Dominici M, Le Blanc K, Mueller I, Slaper-Cortenbach I, Marini F, Krause D (2006). Minimal criteria for defining multipotent mesenchymal stromal cells. The International Society for Cellular Therapy position statement. Cytotherapie.

[5] Rehman J, Traktuev D, Li J, Merfeld-Clauss S, Temm-Grove CJ, Bovenkerk JE (2004). Secretion of angiogenic and antiapoptotic factors by human adipose stromal cells. Cirulation.

[6] Anderson P, Souza-Moreira L, Morell M, Caro M, O’Valle F, Gonzalez-Rey E (2013). Adipose-derived mesenchymal stromal cells induce immunomodulatory macrophages which protect from experimental colitis and sepsis. Gut.

[7] Kilroy GE, Foster SJ, Wu X, Ruiz J, Sherwood S, Heifetz A (2007). Cytokine profile of human adipose-derived stem cells: expression of angiogenic, hematopoetic, and pro-inflammatory factors. J Cell Physiol.

[8] Trojahn Kolle SF, Olivieri RS, Glovinski PV, Elberg JJ, Fischer-Nielsen A, Drzewiecki KT (2012). Importance of mesenchymal stem cells in autologous fat grafting: a systematic review of existing studies. J Plast Surg Hand Surg.

[9] Zuk PA (2010). The adipose-derived stem cell: looking back and looking ahead. Mol Biol Cell.

[10] Gonzalez MA, Gonzalez- Rey E, Rico L, Buescher D, Delgado M (2009). Treatment of experimental arthritis by inducing immune tolerence with human adipose-derived mesenchemal stem cells. Arthritis Rheum.

[11] Lindroos B, Aho KL, Kuokkanen H, Räty S, Huhtala H, Lemponen R (2010). Differential gene expression in adipose stem cells cultured in allogeneic human serum versus fetal bovine serum. Tissue Eng.

[12] Khasawneh RR, AI Sharie AH, Abu-El Rub E, Serhan AO, Obeidat HN (2019). Addressing the impact of different fetal bovine serum percentages on mesenchymal stem cells biological performance. Mol Biol Rep.

[13] Sowa Y, Mazda O, Tsuge I, Inafuku N, Kishida T, Morimoto N (2022). Roles of adipose-derived stem cells in cell-based therapy: current status and future scope – a narrative review. Digest Med Res.

[14] Gstraunthaler G (2003). Alternatives to the use of fetal bovine serum: serum-free cell culture. ALTEX.

[15] Dahl JA, Duggal S, Coulston N, Millar D, Melki J, Shahdadfar A (2008). Genetic and epigenetic instability of human bone marrow mesenchymal stem cells expanded in autologous serum or fetal bovine serum. Int J Dev Biol.

[16] Shahdadfar A, Fronsdal K, Haug T, Reinholt FP, Brinchmann JE (2005). In vitro expansion of human mesenchymal stem cells: choice of serum is a determinant of cell proliferation, differentiation, gene expression and transcriptome stability. Stem Cell.

[17] Bourin P, Bunnel BA, Casteilla L, Dominici M, Katz AJ, March KL (2013). Stromal cells from the adipose tissue-derived stromal vascular fraction and culture expanded adipose tissue-derived stromal/stem cells: a joint statement of the International Federation for Adipose Therapeutics and Science (IFATS) and the International Society for Cellular Therapy (ISCT). Cytotherapy.

[18] Kenneth JL, Schmittgen TD (2001). Analysis of relative gene expression data using real-time quantitative PCR and the 2-ΔΔCT method. Methods.

[19] Safwani W, Makpol S, Sathapan S, Chua KH (2013). Alteration of gene expression levels during osteogenic induction of human adipose derived stem cells in long-term culture. Cell Tissue Bank.

[20] Requicha JF, Viegas CA, Albuquerque CM, Azevedo JM, Reis RL, Gomes ME (2012). Effect of anatomical origin and cell passage number on the stemness and osteogenic differentiation potential of canine adipose-derived stem cells. Stem Cell Rev and Rep.

[21] König MA, Canepa DD, Cadosch D, Casanova E, Heinzelmann E, Rittirsch D (2016). Direct transplantation of native pericytes from adipose tissue: a new perspective to stimulate healing in critical size bone defects. Cytotherapy.

[22] Kim WS, Park BS, Sung JH, Yang JM, Park SB, Kwak SJ (2007). Wound healing effect of adipose-derived stem cells: a critical role of secretory factors on human dermal fibroblasts. J Dermatol Sci.

[23] Schofield R (1978). The relationship between the spleen colony-forming cell and the haemopoietic stem cell. Blood Cell.

[24] Sterodimas A, De Faria J, Correa WE, Pitanguy I (2009). Tissue engineering in plastic surgery. Ann Plast Surg.

[25] Plaks V, Kong N, Werb Z (2015). The cancer stem cell niche: how essential is the niche in regulating stemness of tumor cells?. Cell Stem Cell.

[26] Voog J, Jones DL (2010). Stem cells and the niche: a dynamic duo. Cell Stem Cell.

[27] Fuchs E, Tumbar T, Guasch G (2004). Socializing with the neighbors: stem cells and their niche. Cell.

[28] Yang YI, Kim HI, Choi MY, Son SH, Seo MJ, Seo JY (2010). Ex vivo organ culture of adipose tissue for *in situ* mobilisation of adipose-derived stem cells and defining the stem cell niche. J Cell Physiol.

[29] Kfoury Y, Scadden DT (2015). Mesenchymal cell contributions to the stem cell niche. Cell Stem Cell.

[30] Kim WS, Park BS, Kim HK, Park JS, Kim KJ, Choi JS (2008). Evidence supporting antioxidant action of adipose-derived stem cells: protection of human dermal fibroblasts from oxidative stress. J Dermatol Sci.

[31] Kuhbier JW, Bucan V, Reimers K, Strauss S, Lazaridis A, Jahn S (2014). Observed changes in the morphology and phenotype of breast cancer cells in direct co-culture with adipose-derived stem cells. Plast Reconstr Surg.

[32] Oh J, Lee YD, Wagers AJ (2014). Stem cell aging: mechanisms, regulators and therapeutic opportunities. Nat Med.

[33] Bucciantini M, Giannoni E, Chiti F, Baroni F, Formigli L, Zurdo J (2002). Inherent toxicity of aggregates implies a common mechanism for protein misfolding diseases. Nature.

[34] Joshkon A, Heim X, Dubrou C, Bachelier R, Trabpulsi W, Stalin J (2020). Role of CD146 (MCAM) in physiological and pathological angiogenesis – contribution of new antibodies for therapy. Biomedicines.

[35] Phetfong J, Tawonsawatruk T, Seenprochawong K, Srisarin A, Isarankura-Na-Ayudhya C, Supokawej A (2017). Re-using blood products as an alternative supplement in the optimisation of clinical-grade adipose-derived mesenchymal stem cell culture. Bone Joint Res.

[36] Stahle MU, Brandhost D, Korsgren O, Knutson F (2011). Pathogen inactivation of human serum facilitates its clinical use for islet cell culture and subsequent transplantation. Cell Transplant.

[37] Tallheden T, Van der Lee J, Brantsing C, Mansson JE, Sjögren-Jansson E, Lindahl A (2005). Human serum for culture of articular chondrocytes. Cell Transplant.

[38] Kobayashi T, Watanabe H, Yanagawa T, Tsutsumi S, Kayakabe M, Shinozaki T (2005). Motility and growth of human bone-marrow mesenchymal stem cells during ex vivo expansion in autologous serum. J Bone Joint Surg.

